# The Appraisal of Self-Care Agency Scale-Revised (ASAS-R): Reliability and Validity among Portuguese Medical Students

**DOI:** 10.3390/ijerph191710848

**Published:** 2022-08-31

**Authors:** Lídia Oliveira, Andreia Teixeira, Ivone Duarte

**Affiliations:** 1 Faculty of Medicine, University of Porto, 4200-319 Porto, Portugal; 2 Department of Community Medicine, Information and Decision in Health (MEDCIS), Faculty of Medicine, University of Porto, 4200-319 Porto, Portugal; 3 Center for Health Technology and Services Research (CINTESIS), University of Porto, 4200-319 Porto, Portugal; 4 ADiT-LAB, Instituto Politécnico de Viana do Castelo, Rua Escola Industrial e Comercial Nun’Álvares, 4900-347 Viana do Castelo, Portugal

**Keywords:** self-care, medical students, medical education, questionnaire, psychometrics

## Abstract

Self-care agency plays an important role in an individual’s health. Considering the particularities of their education, it can be a protective factor for the mental health of medical students. This study aims to validate the Portuguese version of the Appraisal of Self-care Agency Scale-Revised (ASAS-R) in Portuguese medical students. A cross-sectional, quantitative, and analytical online study was conducted between 23 April 2021, and 15 July 2021. Exploratory factor analysis (EFA) was performed to test the internal structure of the ASAS-R. Correlations with the SHS (Subjective Happiness Scale), SWLS (Satisfaction with Life Scale), and MHI (Mental Health Inventory) were calculated using Pearson’s coefficient so as to test convergent validity. Internal consistency was evaluated using inter-item correlation, corrected item-total correlation, and Cronbach’s alpha. The total sample included 781 individuals. EFA yielded a 3-factor solution accounting for 53.9% of the explained variance. Pearson’s coefficients obtained between the 3 factors of ASAS-R and the total scores of the 3 construct-related scales demonstrated adequate convergent validity. Total Cronbach’s alpha value was 0.85, while the Cronbach’s alpha of the three factors was 0.81, 0.55, and 0.83, respectively. This study demonstrated that ASAS-R is a valid and reliable instrument for measuring self-care agency among Portuguese medical students.

## 1. Introduction

The concept of self-care is defined by Dorothea Orem in the nursing theory of self-care deficit. According to the author, self-care can be defined as the “practice of activities that individuals start and perform for their benefit, for the maintenance of life, health, and well-being” [1] (p. 19). The ability for one’s self-care can be innate (to fulfil one’s basic needs) but also learned (ability and responsibility to take initiative and function effectively in the development of its potential for health and well-being, with or without the help of health professionals) [1].

If the individual possesses the ability and the self-determination to play the role of self-care agent, the self-care agency or deliberate self-care action comprehends the capability to assess their health and well-being and take appropriate attitudes or actions of self-care. The self-care agency, according to Orem, is defined as “the acquired, complex capacity to meet the requirements to take care of oneself, regulating life processes, maintaining or promoting integrity, structure, and functioning, as well as one’s development and promotion of well-being” [1] (p. 20).

Self-care agency is a complex concept that can be described as dependent on three types of personal trait components: (1) foundational traits, (2) power components/enabling traits, and (3) operational traits [1,2,3,4,5]. The first relates to the ability to perform any deliberate action—not just self-care-related actions—and relies on the individuals’ sensation, perception, memory, and orientation. The second refers to the ability to take deliberated actions related specifically to the engagement in self-care, and it comprehends the individual self-care skills, knowledge, and energy. The third comprehends the most immediate set of abilities required to perform self-care operations, such as the ability to search about the significant factors for one’s self-care, the ability to make decisions and judgments about one’s self-care, and taking action to meet one’s self-care needs.

The biopsychosocial context factors, previous life experiences, and life expectations influence one’s self-care agency. Thus, there are determining factors of self-care agency that vary according to the individual’s (1) physical developmental level (functional capacity, activity tolerance); (2) cognitive developmental level (knowledge about healthy lifestyle and disease, ability to seek information and make decisions); (3) psychological development level (proactivity and motivation, previous experiences, resilience, adaptability, satisfaction with life, emotional/mental state); (4) sociodemographic characteristics (age, sex, marital status, employment situation); (5) social context (support network perceived and used); (6) spiritual beliefs and cultural context; and (7) economic and financial condition [1,6,7,8,9]. The individual’s self-care agency enables oneself to carry out self-care actions that have a direct impact on the promotion of one’s well-being, health, and disease management [4].

Throughout their academic journey, medical students experience intense stressful events and must deal with the distressful aspects of their education, particularly its long duration, a large amount of time dedicated to studying activities, the demand for optimal academic results, among others. These often give rise to compromises in their personal and social lifestyles that may lead to a deterioration of students’ mental health [10]. In fact, lack of motivation, little involvement in leisure activities, lack of emotional support, and academic overload seen in some medical students are correlated with a higher incidence of mental health problems [11].

Several studies demonstrate a high prevalence of depression or depressive symptoms, anxiety, suicidal ideation, psychological distress, and burnout in medical students worldwide [12,13,14,15,16,17,18]. Studies comparing the prevalence of depressive symptoms in medical students before and throughout the medical course show an increase of symptoms during this period [14]. Furthermore, systematic reviews demonstrate higher levels of burnout and psychological distress among medical students when compared to students of other academic fields [16,18,19].

In Portugal, only a few studies assess the mental health and well-being of medical students. One study states that a sizeable proportion of medical students perceive important levels of pathologic stress. The latter was associated with poor sleeping habits and dissatisfaction with social life and academic experience. Furthermore, the levels of burnout and mental illness among Portuguese medical students seem to be greater than the ones observed in students of other academic fields [20]. Experiencing such mental health problems throughout medical school can lead to adverse repercussions in their future professional life, particularly during the first years of medical practice [21].

There are few publication papers regarding self-care and self-care agency in medical students or its correlations with well-being and mental illness. A study developed in Yale University [22] found that a significant proportion of medical students neglect self-care and only half of the students sought appropriate medical care when needed. Additionally, one study conducted in the United States of America among medical students [23] concluded that self-care behaviors evinced a protective effect on the relationship between stress perception and quality of life. Moreover, at the University of Chile, an educational program about self-care [24] attended by medical students led to a decrease in burnout levels and perceived stress and an increase in well-being, mindfulness, and resilience.

In the literature, there are several instruments that can be employed to measure self-care agency: (1) the exercise of self-care agency scale [25], (2) the Denyes’ self-care agency instrument [26], (3) the perception of self-care agency questionnaire [27], (4) the self as a carer inventory [28], (5) the appraisal of self-care agency scale [29] and (6) its revised version [30], and (7) the self-care activities screening scale [31]. However, none of the instruments mentioned above holistically assess the three types of personal trait components that characterize the concept of self-care agency. Thus, different complementary instruments should be considered to measure self-care for a more global understanding of this concept [2,3,4].

The most widely employed instruments of self-care agency measurement are the Appraisal of Self-care Agency Scale (ASAS) [29] and its revised version (ASAS-R) [30]. The concept of self-care agency mentioned above constitutes the theoretical basis for the ASAS-R. The ASAS [29] was originally developed with the purpose to create an instrument that could be generally applied to adults (age > 18 years old), in several states of health. The original scale included 24 items scored on a Likert type scale ranging from 1 (totally disagree) to 5 (totally agree). Subsequently, Souza et al. [30], in a refinement study of the ASAS, showed that the 15-item scale (ASAS-R) extracted from the original scale seemed to be more efficient in measuring self-care agency among individuals from the general population. The ASAS-R is a brief, valid, and reliable instrument of easy applicability to measure self-care agency in individuals of the general population. This instrument was previously used and validated in some populations and countries, namely in individuals from the general population [32] and diabetic individuals in Brazil [33], as well as in elderly individuals in China [34] and Spain [35].

Considering the prevalence of mental health problems among medical students and the scarce literature produced about self-care agency in this particular population, this paper aims at validating the Portuguese version of the Appraisal of Self-care Agency Scale-Revised [32] among medical students in mainland Portugal.

## 2. Materials and Methods

### 2.1. Study Design, Participants, and Sample Size

This is a cross-sectional, quantitative, and analytical study that evaluates the psychometric proprieties of the ASAS-R among medical students in Portugal, seeking its validation in Portuguese context. Data were collected through the application of an online questionnaire in medical students at all medical schools in continental Portugal.

This article follows the Strengthening the Reporting of Observational Studies in Epidemiology (STROBE) guidelines. Participants provided their free and informed consent in the online questionnaire itself. The study began after the approval of the Ethics Committee of São João Center (Ref CE 388-20 on 16 October 2020) and was conducted following the ethical principles enshrined in the Declaration of Helsinki (2013) and the Convention for the Protection of Human Rights and Dignity of the Human Being regarding the Application of Biology and Medicine (2001).

Cohen [36] states that in psychometric studies, the sample size (n) should be at least 300. Nunnally [37] states that the minimum sample size should be *n* = 5 k when the researcher intends to analyze k variables (k > 15), and *n* = 10 k when k < 15. The sample of this study included 781 individuals and therefore attains both authors’ recommendations.

### 2.2. Instruments

The questionnaire consists of 5 sections. In Section I, data was collected for the sociodemographic characterization (gender, age, marital status, among others). The remaining sections are composed of the following instruments.

#### 2.2.1. Appraisal of Self-Care Agency Rating Scale-Revised (ASAS-R)—(Section II)

Version translated into Portuguese and validated in medical students in Brazil by Damásio [32], of the Sousa’s original ASAS-R scale [30]. This instrument is a 15-item scale that appraises the enabling traits (power components) of self-care agency and uses a Likert type scale ranging from 1 (totally disagree) to 5 (totally agree), with total scores ranging from 15 to 75, and higher scores indicating greater self-care agency. Four items of the scale are worded negatively and are reversely scored. The 15 items are distributed by 3 factors that explain 53.5% of the total variance: Factor I—having the capacity for self-care; Factor II—developing the capacity for self-care; and Factor III—inability to self-care. The Portuguese version of the ASAS-R has Cronbach’s alpha values for factors I, II, and III of 0.84, 0.81, and 0.79, respectively [32].

#### 2.2.2. Subjective Happiness Scale (SHS)—(Section III)

A version was translated into Portuguese and validated by Paola Spagnoli, António Caetano, and Ana Junça-Silva [38] from the original version by Lyubomisrky and Lepper [39]. The scale consists of 4 items scored on a scale from 1 to 7 and accesses one’s perception of happiness as well as compared to peers. In the first two items, the subjects are asked to evaluate how they feel about themselves plus compared to others (1 = not a very happy/less happy person to 7 = a very happy/happier person). The third and fourth items offer short descriptions of happy and unhappy individuals and survey how well does each characterization describe the respondent (1 = not at all to 7 = a great deal). The fourth item is negatively worded and, therefore, is reversely scored. The total score is equal to the mean value of the 4 items’ scores and ranges from 1 to 7 with a higher value indicating greater self-perception of happiness. The Portuguese version of the SHS has a good internal consistency with a Cronbach’s alpha value of 0.77 [38].

#### 2.2.3. Satisfaction with Life Scale (SWLS)—(Section IV)

Version translated to Portuguese and adapted by Neto [40], revalidated by António Simões [41]. It is an instrument composed of 5 items that aims to assess the individual’s perception of the level of satisfaction with his/her life. The version used in this study was the version revalidated by Simões in 1992 [41]. Each item is scored on a Likert scale from 1 to 5 (1—strongly disagree, 2—disagree, 3—neither agree nor disagree, 4—agree, 5—strongly agree). The score ranges from 5 to 25, with higher values indicating greater satisfaction with life. The Portuguese version of the SWLS is a reliable instrument with a Cronbach’s alpha value of 0.77 [41].

#### 2.2.4. Mental Health Inventory (MHI)—(Section V)

A version was translated to Portuguese and validated by José Luís Pais Ribeiro [42] of the original version by Veit and Ware [43]. MHI is an instrument that evaluates mental health issues such as anxiety, depression, behavioural control, positive affect, and general distress in general population. In effect, MHI was developed to measure differences in mental health indicators not only among those diagnosed with psychopathological disorders, but also in healthy individuals. The instrument includes 38 items that are distributed by two main factors: psychological distress and psychological well-being; psychological distress results from the sum of the anxiety, depression, and loss of emotional/behavioural control subscales, while psychological well-being results from the sum of the general positive affect and emotional ties subscales. Each item is rated with a maximum of 5 to 6 values by the participant and the total score consists of the result of the sum of the values of the items and must be converted from 0 to 100, with higher values being associated with better mental health. The Portuguese version of the MHI is a valid and reliable instrument and has a Cronbach’s alpha value of 0.96 [42].

### 2.3. Procedure and Data Collection

Initially, a pre-test of the following questionnaire was conducted on a convenience sample of 10 students to identify possible difficulties in understanding the instructions or items. There were not reported any interpretation or format issues of the online survey. Subsequently, an online questionnaire was disclosed to all Medical Schools in continental Portugal in collaboration with their student associations and administrative services. The online survey made use of a volunteer sample and was available for over 12 weeks (23 April 2021, to 15 July 2021).

### 2.4. Data Analysis and Statistical Methods

The data analysis was performed using SPSS^®^ v.27 (SPSS Inc., Chicago, IL, USA). Nominal variables were described by absolute and relative frequencies, *n* (%). Normally continuous variables were represented using mean and the respective standard deviation, M (sd). Non-normal continuous variables or ordinal variables were summarized by median and interquartile interval, Med (Q_1_; Q_3_). The normality of the variables was verified by observation of the respective histograms.

Two types of validity of the adapted ASAS-R to Portuguese language were analyzed: validity of the internal structure and convergent validity. To test the internal structure validity, the technique of principal component analysis was applied with varimax rotation and a cut-off of absolute value higher than 0.4 for loading factors. The applicability of principal component analysis will be tested by calculating the Kaiser-Meyer-Olkin—KMO (measure of sampling adequacy index)—the recommendations indicate that this index should have values higher than 0.6 to apply the principal component analysis. The Bartlett test was also used to compare the correlation matrix with the identity matrix. Regarding the convergent validity of the ASAS-R, correlations were calculated with scales that are theoretically related with ASAS-R: SHS, SWLS, and MHI using the Pearson’s coefficient. It is expected that the scores of Factors I—having the capacity for self-care and II—developing the capacity for self-care have a positive correlation coefficient with the scores of the SHS), SWLS and the MHI. On the other hand, Factor III—Inability to self-care is expected to have a negative correlation coefficient with the scores of the 3 scales mentioned above [32,35,44,45,46].

For reliability testing of the adapted ASAS-R to Portuguese language, the internal consistency was evaluated via inter-item correlation (mean of the inter-item correlation), corrected item total correlation and Cronbach’s alpha because internal consistency implies reliability. The mean of the inter-item correlation should be greater than 0.4 to indicate adequate internal consistency. The corrected item-total correlation was ‘very good’ if the values are between 0.4 and 1.0; ‘good, can improve’ if between 0.3 and 0.39; ‘sufficient but needs improvement’ if between 0.2 and 0.29; and ‘weak, reject or revise’ if between −1.0 and 0.19. Values of Cronbach’s alpha above 0.7 were considered acceptable.

There are no missing values to be handled, as the online survey was composed of items of mandatory answer. *p* values < 0.05 were considered significant.

## 3. Results

### 3.1. Participant Characterization

Of the total 10,424 students enrolled in medical education in Portugal mainland throughout the period of the data collection, a sample of 781 responded to the online questionnaire (response rate: 7.5%). Response rates per medical school varied from 4–14.1% (Faculty of Medical Sciences of Nova Medical School and Faculty of Medicine of the University of Porto, respectively). The detailed sample’s sociodemographic description and health status are presented in Table 1.

### 3.2. Descriptive Statistics of the ASAS-R

Table 2 presents the descriptive statistics of each of the 15 items of the ASAS-R among the 781 individuals included in this study. The items of the Factors I—Having power for self-care (items 1, 3, 8, and 10) and II—Developing power for self-care (items 7, 9, 12, and 13) showed mean scores that varied from 3.47 to 4.03, indicating global positive scores on one’s self-perception of having and developing the ability for self-care. Nonetheless, in the items of the Factor III—Lacking power for self-care (items 4, 6, 11, 14, and 15), means scores varied from 2.35 to 3.32, suggesting that, despite the general positive scores obtained in the previous two factors, the students acknowledge lacking energy, time, and organization for taking care of themselves.

### 3.3. Validation of the Internal Structure of the ASAS-R

The validation of the internal structure of the 15 items of the ASAS-R scale was analyzed by the main component analysis method. Both the KMO measure of sampling adequacy of 0.893 and Bartlet test of sphericity significance level (*p*-value < 0.001) confirmed that it is appropriate to apply this method to this sample. The main component analysis yielded a three-factor solution, which attained the criteria of eigenvalues greater than one, which accounts for 53.9% of the explained variance. In effect, this analysis established a similar factor structure to the one found in the literature. Hence, the 15 items were divided into three main factors: Factor I—Having power for self-care (items 1, 2, 3, 5, 8, and 10); Factor II—Developing power for self-care (items 7, 9, 12, and 13); and Factor III—Lacking power for self-care (items 4, 6, 11, 14, and 15). The loading factors of each item that are adequate (factor loading > 0.4) to the factor structure yielded are represented in Table 3.

### 3.4. Convergent Validity

Pearson’s coefficient was calculated between the SWLS, SHS, and MHI total scores and each factor of the ASAS-R to evaluate convergent validity. Table 4 presents the discriminated correlation coefficients between the stated variables. A positive correlation was found between the factors “having power for self-care” and “developing power for self-care” and the SWLS, SHS, and MHI total scores. Furthermore, a negative correlation was found between the mentioned scale scores and the factor “Lacking power for self-care”. The significant correlation coefficients found in this analysis supported adequate convergent construct related validity of the ASAS-R among Portuguese medical students.

### 3.5. Internal Consistency

The overall estimate of reliability of the ASAS-R was a Cronbach’s alpha value of 0.85. Cronbach’s alpha was also calculated to evaluate the internal consistency of each of the factors. Table 5 presents the inter-item correlation (mean), the corrected item-total correlation, and the Cronbach’s alpha in case the item in question was excluded. The factors “Having power for self-care” and “Lacking power for self-care” showed an inter-item correlation mean greater than 0.4 (0.42 and 0.49, respectively). However, the factor “Developing power for self-care” revealed an inter-item correlation mean less than 0.4 (0.24). In the factors “Having power for self-care” and “Lacking power for self-care”, all items have a particularly good item-total correlation. In the “Developing power for self-care” factor, items 9 and 13 have a corrected item-total correlation ‘good, can improve’ and item 7 presents a corrected item-total correlation ‘sufficient but needs improvement’. In all three factors, removing any item would reduce Cronbach’s alpha value of the respective factor (0.81, 0.55, and 0.83, respectively). In this study, the Cronbach’s alpha value of each instrument used to explore convergent validity of the ASAS-R was also calculated (SHS—0.86; SWLS—0.83; MHI—0.97). The reported values confirmed adequate internal consistency and, consequently, reliability of the mentioned scales.

## 4. Discussion

The present study is the first study to analyze the psychometric properties of the Portuguese version of the ASAS-R among Portuguese medical students. The findings support the reliability and validity of the Portuguese version of the ASAS-R in Portuguese medical students.

The exploratory factor analysis revealed three factors with an eigenvalue greater than one, which accounted for 53.9% of the explained variance. The three-factor solution obtained is similar to the one reported by previous validation studies [30,32,33,34,35]. Nonetheless, items 6 and 8, which according to the original validation study by Souza et al. [30] were expected to load in Factors I and II, respectively, in the present study loaded in Factors III and I, respectively. These findings are in line with the results shown by Damásio et al. [32]. Furthermore, in the study conducted by Stacciarini et al. [33], the Lagrange multiplier test suggested the reallocation of item 8 in factor I. The authors cannot find a comprehensive explanation for these differences. Nonetheless, given the similarities between factor measures and items, such differences are unlikely to compromise the theoretical foundation of the ASAS-R [32]. Most previous validation studies [30,32,33,34,35] performed a Confirmatory Factor analysis in the addiction of the EFA. Nevertheless, in the present study, the authors considered EFA to be an adequate method to study the internal structure validity as this is the first research that applies this instrument in Portugal.

Regarding convergent validity, previous validation studies made use of Pearson’s correlations between the ASAS-R and construct-related scales, such as, the Health-Promoting Lifestyle Profile II (HPLP-II) [47], SWLS [40], SHS [39], Short Form Health Survey 12 Item v2 (SF-12v2) [48], Short Form Health Survey 36 Item v2 (SF-36v2) [49], and the exercise of self-care agency scale (ESCA) [25]. The present study made use of a similar set of construct-related scales and confirmed adequate convergent validity. Positive correlations were found between the Factors I “having power for self-care” and II “developing power for self-care” and the SWLS, SHS, and MHI scores. This finding suggests that greater ability for self-care is associated with greater satisfaction with life, perception of happiness, and mental health, as reported by Damasio et al. [32]. In fact, several studies support the hypothesis that self-care agency has an important role in both physical and mental health [32,35,45,46]. Such can be explained by the fact that individuals with greater self-care agency are more likely to care for themselves, perform self-care actions, and adopt healthy behaviors [30], that positively affect their physical and mental health. Furthermore, several studies suggest that self-care agency is associated with well-being and satisfaction with life [32,44,50,51]. Comprehensively, this association might be related to the positive influence of self-care actions and health-promoting behaviors on physical and mental health, quality of life, and, consequently, on well-being and satisfaction with life.

In the present study, Factor I “having power for self-care” had higher correlation values with the total scores of SHS, SWLS, and MHI than Factor II “developing power for self-care”. These findings are in line with the results reported by Damasio et al. [32], and suggest that “having power for self-care” is a more important factor influencing happiness, satisfaction with life, and mental health than “developing power for self-care”.

On the other hand, Factor III “lacking power for self-care” showed a negative correlation with all 3 comparative scales’ total scores. This association corroborates the results reported in previous studies, suggesting that poor self-care agency or “lacking power for self-care agency” are associated with risk for mental illness [45,46] and low happiness and life satisfaction [32].

The results concerning the analysis of the total internal consistency of the ASAS-R (Cronbach’s alpha = 0.85) and Factors I “Having power for self-care” and III “Lacking power for self-care” (Cronbach’s alphas of 0.81 and 0.83, respectively) were considered satisfactory (>0.7), and similar to the original version [30] and all the subsequent validation studies [32,33,34,35]. However, Cronbach’s alpha value of Factor II “Developing power for self-care” found in this study was lower than 0.7 (0.55), and therefore was considered unsatisfactory [37]. This finding only relates to the value reported by Stacciarini et al. [33] (Cronbach’s alpha = 0.38). The Cronbach’s alpha value of Factor II can be partially explained by the fact that Factor II is composed of a lower number of items (four items) when compared to Factors I and III (six and five items, respectively).

The mean inter-item correlations were adequate (>0.4) for Factors I “Having power for self-care” and III “Lacking power for self-care” (0.42 and 0.49, respectively) but not for the Factor II “Developing power for self-care” (0.24). Nonetheless, a value of mean interitem correlations between 0.15 and 0.5 are considered sufficient and evince satisfactory internal consistency regardless of the number of items [52]. The item-to-total correlations ranged from 0.29 to 0.71. A very good item to total correlation was found among the items of the Factors I and III. However, weaker item to total correlations was found for items 7, 9, and 13 of factor II. Nonetheless, removing either of the items would decrease the Cronbach’s alpha value of Factor II. These findings are similar to the values found by Stacciarini et al. The authors report weak item to total correlations for items 4, 9, and 13 (values below 0.3), as well as unsatisfactory Cronbach’s alpha and item-to-factor values for factor II.

The present study concluded that the ASAS-R is a valid and effective instrument to measure self-care agency among Portuguese medical students. With this measuring instrument, further research can be conducted to: characterize self-care agency patterns among this particular population; explore determinant factors of the individual’s self-care agency; reveal correlations between self-care agency and pertinent variables, such as, academic achievement, quality of life, burnout, resilience, incidence of physical and mental illnesses, among others; and to study the impact of integrating self-care agency awareness in the academic curriculum, on the students’ self-care ability, as well as its repercussions on health, personal, and academic life indicators.

Although the present study presented valuable results that confirmed the adequacy, validity, and reliability of the ASAS-R among Portuguese medical students, several limitations must be taken into consideration. The questionnaire was divulged in an online form; thus, it was not possible to ensure that the participants were in fact medical students. Moreover, sample representativity might not have been achieved as the response rate was 7.5% and the response rate per medical school varied from 4–14.1%. Although there is a large gap between the percentage of male and female participants (17.5% and 82.5%, respectively), these findings are consistent with the usual gender distribution of Portuguese medical students (female predominance of 72–65%) [53]. Nonetheless, the included sample size was considered adequate to conduct the psychometric analysis of the Portuguese version of the ASAS-R. Additionally, during the period of the data collection, students were still facing restrictions and concerns due to the COVID-19 pandemic. Such context could have affected the answers to the items of the instruments applied as the pandemic represented a stressful event in their personal and academic lives [54]. Similarly, the data collection period included the final exams season, and therefore some responses could have been influenced. In fact, the exams season represents a very stressful time in the lives of medical students, and can be experienced as a period of personal and social privation characterized by prioritization of study time over leisure and self-care actions [55]. Furthermore, the transversal nature of this study did not allow us to identify and explore the mechanisms behind the casual relation between self-care agency and mental health, happiness, and satisfaction with life. Further research based on longitudinal studies can be done to enhance knowledge on this matter. Additionally, the previous validation studies of the ASAS-R were not based solely on medical students, hampering the comparison of the results of the present work with previous findings. Finally, though some students have reported chronic illness, the data was collected through an open answer. The low number of responses and the high response heterogeneity did not allow a comparison between groups or explore the knowledge about self-care agency related to healthcare or illness. Further research can be conducted to better characterize the relationship between self-care agency and prevalent illnesses among medical students, such as depression, burnout, and anxiety, among others.

## 5. Conclusions

This study is the first to analyze the psychometric properties of the Portuguese version of the ASAS-R amongst Portuguese medical students. The results evince that the ASAS-R is a valid and reliable instrument to measure self-care agency among these individuals. Exploratory factor analysis revealed a three-factor-solution consistent with the previous validation studies. Reliability analysis of the ASAS-R and its factor structure was considered adequate, except for factor II “developing power for self-care.” The test for convergent validity showed correlations in the expected direction between the SHS, SWLS, and MHI total scores, and all three factors of the ASAS-R. The present study is expected to contribute to further research on self-care agency among medical students. As discussed, self-care agency plays a significant role in physical and mental health, well-being, and satisfaction with life. Taking into consideration that mental health problems are common in medical students, the application of the ASAS-R can provide greater insight into the relationship between mental health problems and self-care agency. Furthermore, future recommendations can be formulated to improve self-care agency among medical students and, therefore, lower the likelihood of mental health issues.

## Figures and Tables

**Table 1:** Distribution of sociodemographic variables (*n* = 781).

Variables	*n* (%)
Sex, *n* (%)	
Male	137 (17.5)
Female	644 (82.5)
Age (Years), Mean (Q_1_; Q_3_)	22 (20; 24)
Medical School, n (%)	
Faculty of Medicine of the University of Porto	237 (30.3)
Faculty of Medicine of the University of Lisbon	115 (14.7)
Faculty of Medicine of the University of Coimbra	96 (12.3)
Faculty of Medicine of the University of Minho	89 (11.4)
Institute of Biomedical Sciences Abel Salazar	89 (11.4)
Faculty of Medical Sciences of Nova Medical School	65 (8.3)
Faculty of Health Sciences of the University of Beira Interior	62 (7.9)
Faculty of Medicine and Biomedical Sciences of the University of Algarve	28 (3.6)
Year, *n* (%)	
1st	148 (19.0)
2nd	147 (18.8)
3rd	121 (15.5)
4th	104 (13.3)
5th	130 (16.6)
6th	131 (16.8)
Marital status, *n* (%)	
Single	752 (96.3)
Married or civil union	28 (3.6)
Divorced or Separate	1 (0.1)
Chronic disease, *n* (%)	
No	652 (83.5)
Yes	129 (16.5)

**Table 2:** 15-item ASAS-R’s descriptive statistics (*n* = 781).

Items	Med (Q_1_; Q_3_)	M (sd)
1. As circumstances change, I make the needed adjustments to stay healthy	4 (4; 4)	3.86 (0.80)
2. If my mobility is decreased, I make the needed adjustments	4 (3; 4)	3.72 (0.88)
3. When needed, I set new priorities in the measures that I take to stay healthy	4 (3; 4)	3.80 (0.90)
4. * I often lack energy to care for myself in the way that I know I should	4 (2; 4)	3.32 (1.17)
5. I look for better ways to take for myself	4 (3; 4)	3.69 (0.86)
6. When needed, I manage to take time to care for myself	4 (3; 4)	3.47 (1.01)
7. If I take a new medication, I obtain information about the side effects to better care for myself	4 (3; 5)	3.81 (1.10)
8. In the past, I have changed some of my old habits in order to improve my health	4 (3; 4)	3.78 (0.95)
9. I routinely take measures to ensure the safety of myself and my family	4 (4; 5)	4.03 (0.80)
10. I regularly evaluate the effectiveness of things that I do to stay healthy	4 (3; 4)	3.48 (1.02)
11. * In my daily activities I seldom take time to care for myself	3 (2; 4)	3.24 (1.13)
12. I am able to get information I need, when health is threatened	4 (4; 5)	4.03 (0.82)
13. I seek help when unable to care for myself	4 (3; 4)	3.60 (1.05)
14. * I seldom have time for myself	3 (2; 4)	3.12 (1.14)
15. * I am not always able to care for myself in a way I would like	2 (2; 3)	2.35 (1.10)

* These items are worded negatively and, therefore, the answers were reversely scored.

**Table 3:** Composition of the three factors extracted for the ASAS-R scale with factor loadings (>0.4) of the items, obtained by main component analysis (*n* = 781).

Items	Factor I	Factor II	Factor III
1. As circumstances change, I make the needed adjustments to stay healthy	0.734		
2. If my mobility is decreased, I make the needed adjustments	0.671		
3. When needed, I set new priorities in the measures that I take to stay healthy	0.681		
4. * I often lack energy to care for myself in the way that I know I should			0.642
5. I look for better ways to take for myself	0.548		
6. When needed, I manage to take time to care for myself			0.688
7. If I take a new medication, I obtain information about the side effects to better care for myself		0.662	
8. In the past, I have changed some of my old habits in order to improve my health	0.699		
9. I routinely take measures to ensure the safety of myself and my family		0.561	
10. I regularly evaluate the effectiveness of things that I do to stay healthy	0.569		
11. * In my daily activities I seldom take time to care for myself			0.705
12. I am able to get information I need, when health is threatened		0.676	
13. I seek help when unable to care for myself		0.585	
14. * I seldom have time for myself			0.837
15. * I am not always able to care for myself in a way I would like			0.746

* These items are worded negatively and, therefore, the answers were reversely scored.

**Table 4:** Convergent validity (Pearson’s coefficient).

ASAS-R	SWLS	SHS	MHI
Having power for self-care	0.385 *	0.417 *	0.406 *
Developing power for self-care	0.294 *	0.240 *	0.276 *
Lacking power for self-care	−0.401 *	−0.463 *	−0.618 *

ASAS-R: Self-Care Agency Scale-Revised; SWLS: Satisfaction with life scale; SHS: Subjective happiness scale; MHI: Mental health inventory; * *p* < 0.001.

**Table 5:** Internal consistency of the adapted ASAS-R to Portuguese language (*n* = 781).

Factor/Item	Factor α and Change in α if Item Deleted	Item-to-Total Correlation
Factor I: Having power for self-care	0.81	0.42 ^a^
1. As circumstances change, I make the needed adjustments to stay healthy	0.77	0.62
2. If my mobility is decreased, I make the needed adjustments	0.78	0.55
3. When needed, I set new priorities in the measures that I take to stay healthy	0.76	0.63
5. I look for better ways to take for myself	0.77	0.59
8. In the past, I have changed some of my old habits in order to improve my health	0.79	0.51
10. I regularly evaluate the effectiveness of things that I do to stay healthy	0.79	0.51
Factor II: Developing power for self-care	0.55	0.24 ^a^
7. If I take a new medication, I obtain information about the side effects to better care for myself	0.53	0.29
9. I routinely take measures to ensure the safety of myself and my family	0.47	0.34
12. I am able to get information I need, when health is threatened.	0.42	0.41
13. I seek help when unable to care for myself	0.48	0.33
Factor III: Lacking power for self-care	0.83	0.49 ^a^
4. * I often lack energy to care for myself in the way that I know I should	0.80	0.60
6. When needed, I manage to take time to care for myself	0.80	0.61
11. * In my daily activities I seldom take time to care for myself	0.80	0.59
14. * I seldom have time for myself	0.77	0.71
15. * I am not always able to care for myself in a way I would like	0.79	0.62

α = Cronbach’s alpha; a = inter-item correlation mean; * These items are worded negatively and, therefore, the answers were reversely scored.

## Data Availability

The corresponding author can obtain the exact data.
